# Characteristics of Hourly Extreme Precipitation along the Yangtze River Basin, China during Warm Season

**DOI:** 10.1038/s41598-020-62535-5

**Published:** 2020-03-27

**Authors:** Yong Zhao, Anning Huang, Menyun Kan, Xinning Dong, Xiaojing Yu, Yang Wu, Xindan Zhang, Shuxin Cai

**Affiliations:** 1 Plateau Atmosphere and Environment Key Laboratory of Sichuan Province, School of Atmospheric Sciences, Chengdu University of Information Technology, Chengdu, China 610225; 2 State Key Laboratory State of Severe Weather and Joint Center for Atmospheric Radar Research of CMA/NJU, School of Atmospheric Sciences, Nanjing University, Nanjing, China 210023; 3 Glarun Technology Co., ltd, Nanjing, China 211106; 4 Chongqing Climate Center, Chongqing, China 401147; 5 Institute of Desert Meteorology, China Meteorological Administration, Urumqi, China 830002

**Keywords:** Climate sciences, Natural hazards

## Abstract

Based on the hourly gauge-satellite merged precipitation data with the spatial resolution of 0.1° × 0.1° during 2008–2016, the characteristics of extreme precipitation (EP) diurnal cycle along the Yangtze River Basin (YRB) and their regional and sub-seasonal differences during warm season have been indicated and revealed in this study. Results show that the EP amount (EPA) over most lower reaches of YRB exhibits two diurnal peaks with one in late afternoon and the other in morning, while the EPA over most eastern Tibetan Plateau (the Sichuan Basin and the northern Yunnan-Guizhou Plateau) generally peaks during late afternoon to midnight (midnight to early morning). The afternoon (morning) EPA diurnal peaks over the areas east to 110°E is mainly resulted from the short (long) duration EP events. However, both the short and long duration EP events lead to the nocturnal diurnal peaks and eastward propagating features of EPA over the regions west to110°E. The EP events over the Sichuan Basin generally begin at midnight and mostly peak around 03:00-04:00 Beijing time, and they start earlier and end later with the duration time increased. However, the EP events with short (long) duration over the lower reaches of YRB frequently start and peak in afternoon (early morning) and typically end at around 18:00 (07:00-08:00) Beijing time, and they start later (earlier) and end later with the duration time increased. Meanwhile, the EP frequency (EPF) diurnal cycles over the lower reaches of YRB exhibit obvious sub-seasonal differences in warm season, which show only a morning peak in the pre-Meiyu period, two comparable peaks with one in afternoon and the other in morning during the Meiyu period, and a predominant afternoon peak and a secondary morning peak in the post-Meiyu period, respectively. While the EPF over Sichuan Basin characterized by only one dominant early morning peak during all periods of the warm season exhibits much smaller sub-seasonal differences in the diurnal phase relative to that over the lower reaches of YRB.

## Introduction

Increases in the global surface temperature due to the increased greenhouse gases lead to enhanced water-holding capacity of the atmosphere, increased evaporation and atmospheric moisture^1,2^. The accelerated hydrological cycle^3^ may be affected the frequency and intensity of extreme precipitation (EP) events^4–6^, which can have significant environmental, societal, and even political impacts^7^. Earlier studies have shown that the extreme climate events are often more important to natural and human systems than their mean values^8^.Changes in extreme climate events are greater than in mean climate^9,10^. As one of the most important extreme climate events, the EP has attracted a great deal of attention among the scientific community^11,12^ due to its serious and profound effects on agriculture, natural ecosystems and human society^13–15^. So revealing the characteristics of the EP has become a hot topic for government, public and the climatic research community, which can provide a foundation for disaster prevention and mitigation, and regional water resources security and management^16–18^.

The EP in China is common because of the East Asia monsoon systems and different climate zones due to the large variability of monsoonal climate and the unique distribution of topography with a vast land spanning many degrees of latitude and complex terrain, especially in the Yangtze River Basin (YRB)^9,19^, where frequent floods caused by EP in summer inflict considerable loss of economy and human life^20^. During the past several decades the EP along the YRB in summer has increased by 10–20% every 10 years, and significant increase has happened in the mid-lower reaches of YRB in recent two decades^21,22^. Although a lot of studies have been carried out on EP and obtained some valuable results, the previous studies on EP along the YRB are mostly based on the daily rainfall data and focused on the long term trend of EP in several decades or mainly concentrated on the EP with the duration of only one or more days^17,23–25^. However, the EP has different hydrological impacts depending on the duration of events: the EP with short duration often due to convective storms (i.e. sub-daily) may be treated as proxies for flash floods, multi-day EPs are often considered as proxies for large scale floods^26,27^. Studies have indicated that the greatest increases are likely to occur in the short duration storms lasting less than a day, leading to an increase in the magnitude and frequency of flash floods^28^.

Compared to the EP events with the duration up to a few hours, the EP events at daily and multi-day time scales cannot properly reflect the precipitation intensity and frequency and describe more details of the precipitation processes^29,30^. Recent studies based on the ground gauge observed data have indicated that the hourly EP frequently occurring along the YRB has shown an increasing trend^31–33^.Therefore, it is necessary to provide much more detailed information of EP at sub-daily time scales along the YRB. In addition, due to the inhomogeneous surface landform in the YRB, such as plateau, basin, hills, and plain, may cause complex regional scale diurnal features of EP, the hourly EP over the areas with great altitude differences and complex geographic conditions usually experience a rapid development from its occurrence to peak^34^.

Based on the hourly precipitation product of Climate Precipitation Center Morphing (CMORPH) satellite data merged with the data from automatic weather stations (AWS) in China with high spatial resolution^35,36^, the detailed characteristics of the EP over the YRB in warm season (May to August) with much finer spatial resolution (~10 km) will be revealed at hourly time scale. We will focus on addressing such issues as follows: (1) How is the diurnal variation of EP with different duration along the YRB in warm season? (2) What is the feature of EP processes with different duration along the YRB during warm season in terms of their start, peak and end time? (3) How are the regional and sub-seasonal differences in the diurnal cycle of EP with different duration along the YRB during warm season? Findings of current study may provide the basic features of EP along the YRB in warm season at hourly time scale, which may be helpful to deepen our understanding of the EP processes and further improve the EP prediction.

## Study area, data and method

### Study area

**Figure 1:**
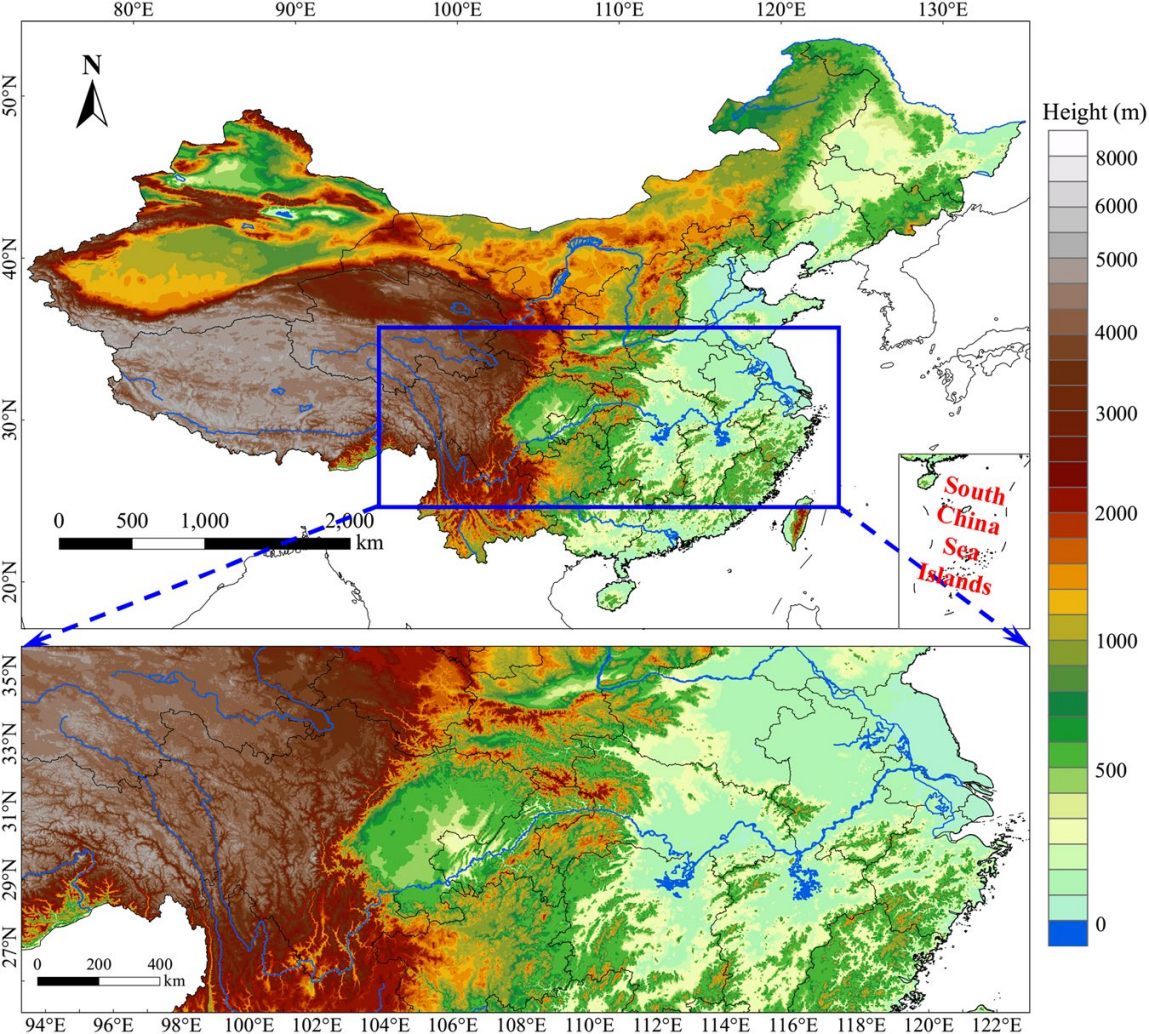
Study domain, shading indicates the terrain height.

**Figure 2:**
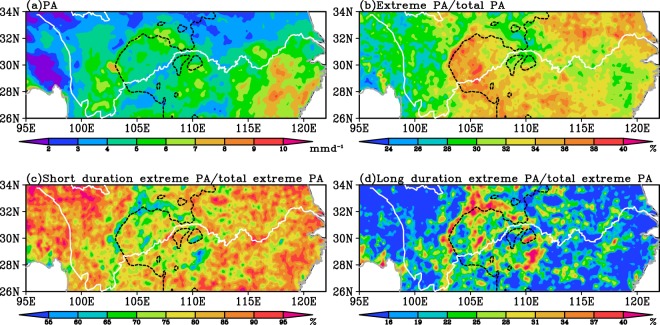
The spatial distribution of PA (**a**), contribution of the TEPA to the total PA (**b**), and contribution of the EPA with short (**b**) and long (**c**) duration to the TEPA in warm season along the Yangtze River Basin averaged over 2008–2016. The white solid line represents the Yangtze River and the dark dashed line is the 1000 m topographic elevation.

This study concentrates on the EP along the YRB (95°E-122°E, 26°N-34°N), China, which is the largest river basin in China covering 19 provinces from west to east with an area of 1.8 × 10^6^km^2^ (Fig. 1). The region is characterized by complex terrain and controlled by the East Asian summer monsoon in warm season (May to August), during which the precipitation amount (PA) accounts for more than 50% of the annual PA (not shown) and the EP frequently occurs^32^. As shown in Fig. 2a, the PA along the YRB in warm season decreases from south to north with relatively larger magnitude located over Sichuan Basin, northern Yunnan-Guizhou Plateau and south of the lower reaches of YRB, where the PA is larger than 5 mm d^−1^. Meanwhile, more than 30% of the total PA over most YRB is contributed by the EP events during warm season, especially in most Sichuan Basin and northern lower reaches of YRB the EP amount (EPA) contributes more than 35% of the total PA (Fig. 2b). The EPA with the short duration (<3 hours) contributes more than 85% of the total EPA (TEPA) over most YRB (Fig. 2c). In addition, the contribution of EPA with long duration (≥3 hours) to the TEPA ranges from 20% to 40% over Sichuan Basin with relatively larger values located along its surrounding mountainous areas (Fig. 2d).

### Data

The hourly gauge-satellite merged precipitation product with the spatial resolution of 0.1° × 0.1° in latitude and longitude^35,36^ during2008–2016 developed by National Meteorological Information Center of China(http://cdc.cma.gov.cn/home.do) is used in this study. This dataset is generated from the gauge observed precipitation at more than 30,000 automatic weather stations in China combined with the CMORPH satellite precipitation product using the improved probability density function-optimal interpolation (PDF-OI) methods originally^37,38^. The cross-validation results suggest that this merged gauge-satellite precipitation product over China generated by the improved PDF-OI method shows much smaller bias, root mean square error and higher spatial correlation relative to the original PDF-OI derived precipitation product and good ability in capturing the varying features of hourly precipitation in heavy weather events^39^.

### Method

The 95^th^ percentile of all historical hourly precipitation time series in warm season during 2008–2016 is regarded as the EP threshold at each grid based on the cumulative frequency distribution (CFD) method^19,32^. Following the previous studies^29,40–42^, the number of hours between the start and end of an EP event without any intermittence during which the precipitation is larger than the EP threshold is defined as the EP duration. The peak time of an EP event is the hour (00:00~23:00 Beijing time) when the precipitation reaches the maximum value within the duration, if the duration is 1 hour, then the start, end and peak time overlap with each other. The EP events are picked out separately based on the duration ranging from 1 to 10 hours at each grid. According to the duration, the EP events are sorted into short duration (<3 hours) type triggered by strong solar heating with the diurnal maximum rainfall around late afternoon and long duration (≥3 hours) type caused by organized rainfall systems closely related to the large scale monsoon circulation rather than isolated convection generally displaying morning diurnal peaks^40–42^. Based on the method mentioned above, we record the basic information of each EP event including the start, end and peak time, and duration, the EPA, EP frequency (EPF), and EP intensity (EPI) at each grid. The EPA is the accumulated EP rainfall divided by the total hours (123 × 9 = 1107 h) in warm season during 2008–2016 at each hour of a day (00:00~23:00Beijing time) on each grid. The EPF is the percentage of the total EP hours to the total hours in warm season during 2008–2016 at each hour of a day on each grid. The EPI equals to EPA/EPF.

In addition, we also adopted the diurnal percentage (DP)^43^ and variation of EPA/EPF/EPI as follows:

1 $$DP=\frac{\mathop{\sum }\limits_{t=0}^{t=23}|({R}_{t}-\overline{R})|}{{R}_{d}}\times 100 \% $$

2 $${D}_{{\rm{t}}}=({R}_{{\rm{t}}}-\bar{R})/\bar{R}$$

where ${R}_{d}$ is the climatic mean daily EPA/EPF/EPI. *R*_*t*_ is the climatic mean hourly EPA/EPF/EPI at the *t*^*th*^ hour of Beijing time in a day. $\bar{R}=\frac{1}{24}\mathop{\sum }\limits_{t=0}^{t=23}{R}_{t}$(${R}_{d}=24\bar{R}$) is the mean hourly (daily) EPA/EPF/EPI. DP measures the EPA/EPF/EPI diurnal variability and ${D}_{{\rm{t}}}$ is the hourly time series of the normalized EPA/EPF/EPI at the *t*^th^ hour ranging from 00:00 to 23:00 Beijing time.

Considering the distinct differences in the climatic mean of the synoptic environment, we divide the warm season into three sub-periods^43^: pre-Meiyu period (1 May to 15 June), Meiyu period (16 June to 15 July), and post-Meiyu period (16 July to August 31) for simplicity. Then the features of EP in different stages of warm season and their sub-seasonal differences are analyzed.

## Results

### Diurnal variation of EP during warm season

**Figure 3:**
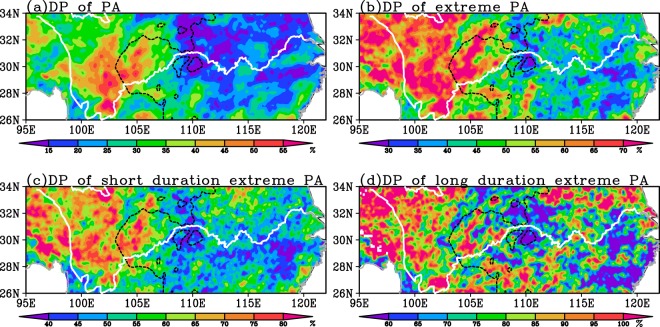
Diurnal percentage of the total PA (**a**), the TEPA (**b**), and the EPA with short (**c**) and long duration (**d**) in warm season along the Yangtze River Basin averaged over 2008–2016. The white solid line represents the Yangtze River and the dark dashed line is the 1000 m topographic elevation.

Figure 3 shows the diurnal percentages of PA along the YRB during warm season. The diurnal percentage of the total PA shows a large center with the magnitude >40% located over the eastern Tibetan Plateau and the western Sichuan Basin, whereas the diurnal percentages in most parts of the middle and lower reaches of YRB are much smaller with the magnitude less than 25% (Fig. 3a). The diurnal percentage of the EPA (Fig. 3b) shows similar distribution features to that of the total PA but with much larger magnitude (around twice as much as that of the total PA over most areas). In the lower reaches of YRB, the EPA diurnal percentages are relatively stronger in the northern region than in the southern region (Fig. 3b). Diurnal percentages of the short duration EPA and TEPA share a similar pattern with comparable magnitude (Fig. 3b,c). The diurnal percentages of the long duration EPA over most YRB show much larger magnitude relative to the TEPA (Fig. 3b,d), especially over the lower reaches of YRB where the diurnal percentage of the long duration EPA is nearly twice as much as that of the TEPA or short duration EPA.

**Figure 4:**
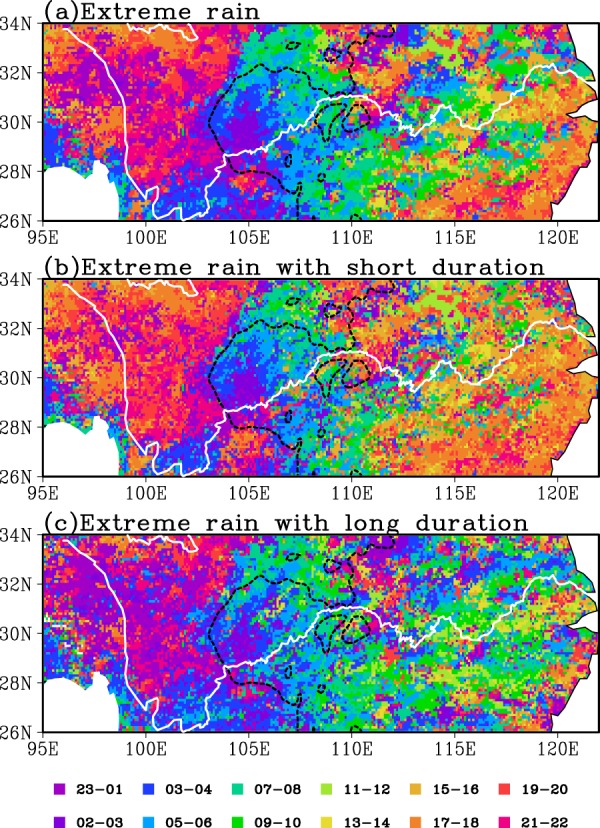
The spatial distribution of the diurnal peak time (Beijing time) for the TEPA (**a**), the EPA with short (**b**) and long duration (**c**) in warm season over 2008–2016. The white solid line represents the Yangtze River and the dark dashed line is the 1000 m topographic elevation.

The diurnal peak time is an important parameter for the precipitation diurnal variation. Previous studies show that the PA in summer exhibit a midnight (late afternoon) diurnal peak over Sichuan Basin (the lower reaches of YRB)^43,44^. As shown in Fig. 4a, the TEPA diurnal peaks over most eastern Tibetan Plateau occur at late afternoon to midnight (17:00~01:00 Beijing time). Meanwhile, it can be noted that the TEPA diurnal peaks in the Sichuan Basin and the northern Yunnan-Guizhou Plateau appear predominantly between 00:00~06:00 Beijing time. The TEPA over most of the downstream of the YRB and the southeast coast shows diurnal peaks between afternoon and early evening, this is very similar to the distribution of the total PA diurnal peak time. In addition, from the eastern Tibetan Plateau through the Sichuan Basin to around 110°E, the EPA peak time clearly shows an eastward-delayed diurnal phase, indicating the eastward propagation of precipitation systems^41,45^. The short duration EPA generally shows afternoon to early evening diurnal peaks over most areas except Sichuan Basin and its southeastern and northern surrounding regions where the short duration EPA shows diurnal peaks during midnight to early morning (Fig. 4b). From Fig. 4c, the long duration EPA over most YRB tends to show nocturnal and morning diurnal peaks. Meanwhile, the diurnal peak time of long duration EPA over the plateau areas (west to 105°E) appears about 2–4 hours later than that of the short duration EPA (Fig. 4b). However, the long duration EPA over most central mid-lower reaches of YRB generally show diurnal peaks at morning to noon (07:00 to 12:00 Beijing time) with 3–4 hours earlier relative to the short duration EPA.

**Figure 5:**
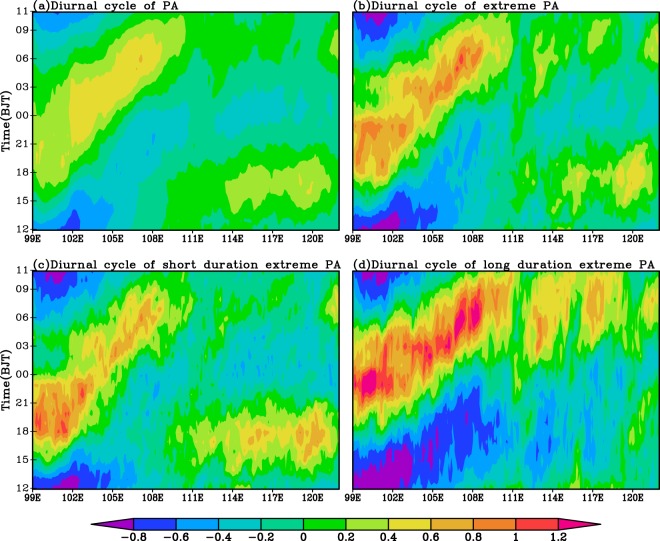
The normalized time-longitude distribution of the climatic mean total PA (**a**) and TEPA (**b**), the EPA with short (**c**) and long (**d**) duration time over 2008–2016 regionally averaged along 26–34 ^o^N in warm season.

From Fig. 5a, the total PA diurnal peaks over the regions west to 110°E show a clear eastward propagation, which begin at 15:00 Beijing time and end at 11:00 Beijing time in the next day. Over the regions east to 110°E the PA shows a predominant diurnal peak in afternoon and a secondary diurnal peak in morning without propagating features. The PA afternoon peaks are mainly caused by the unstable lower layers due to the radiative heating of the surface and the subsequent convergence and rise of water vapor^46^. The nighttime PA maxima over the eastern plateau are likely due to the radiative cooling at the top of the stratus, resulting in instability. The eastward growth of the nighttime precipitation is mainly affected by the clockwise rotation of the southwesterly winds in the lower layers^43,46^.

As shown in Fig. 5b, the TEPA shows very similar distribution to the total PA but with much stronger magnitudes. Comparisons of the distribution of short (Fig. 5c) and long (Fig. 5d) duration EPA and the TEPA (Fig. 5b), the afternoon (morning) diurnal peaks of the TEPA over the areas east to 110°E are largely contributed by the short (long) duration EPA. However, the nocturnal diurnal peaks and eastward propagating features of TEPA over the regions west to 110°E are mainly resulted from both short and long duration EP.

Based on the west-east differences in the TEPA along the YRB shown in Figs. 4a and 5b and the terrain distribution, we highlight two sub-regions of the study region, such as the Sichuan Basin (Reg.1, 102°–110°E, 26°–34°N) and the lower reaches of the YRB (Reg.2, 110°–122°E, 26°–34°N), to indicate the regional differences in the EP diurnal variation between the two sub-regions.

**Figure 6:**
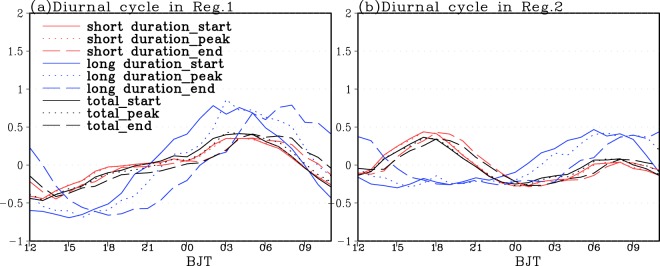
The diurnal cycle of the normalized occurrence frequency at the onset (solid line), peak (dotted line) and end time (dashed line) for the total EP (black) and the EP with short (red) and long (blue) duration time regionally averaged over Sichuan Basin (**a**) and the lower reaches of Yangtze River Basin (**b**) during 2008–2016.

Figure 6 shows the diurnal cycle of the normalized EPF at the onset, peak and end time of EP events with different duration regionally averaged over each sub-region. From Fig. 6a, the EP with short and long duration and the total EP over the Sichuan Basin consistently tends to occur frequently at around 02:00~04:00 Beijing time. The total EPF (TEPF) shows very similar diurnal cycles to the short duration EPF. However, the diurnal cycles of the long duration EPF show much larger diurnal variability. In addition, the long duration EP events frequently occur at around 02:00 Beijing time with peaks at around 03:00 Beijing time and generally end at about 08:00 Beijing time, they take relatively shorter time to reach their peaks after starts and much longer time to ends after peaks. In the lower reaches of YRB, the diurnal variations of short duration EPF also coincide with those of TEPF. The short duration EP events mostly occur in the afternoon, while the long duration EP events typically start around 06:00 Beijing time, peak at 08:00 Beijing time and end at 11:00-12:00 Beijing time. Similar to the EP with long duration over the Sichuan Basin (Fig. 6a), these events also take relatively shorter time to reach the peaks after starts and much longer time to reach the ends after peaks (Fig. 6b). Overall, the frequent occurrences of both short and long duration EP events during midnight to early morning in terms of the starts, peaks and ends of EP events lead to the nocturnal peaks of the TEPF over Sichuan Basin. However, the TEPF over the lower reaches of YRB with double diurnal peaks tends to show a predominant (secondary) peak in afternoon (early morning) due to the frequent occurrences of EP with short (long) duration.

**Figure 7:**
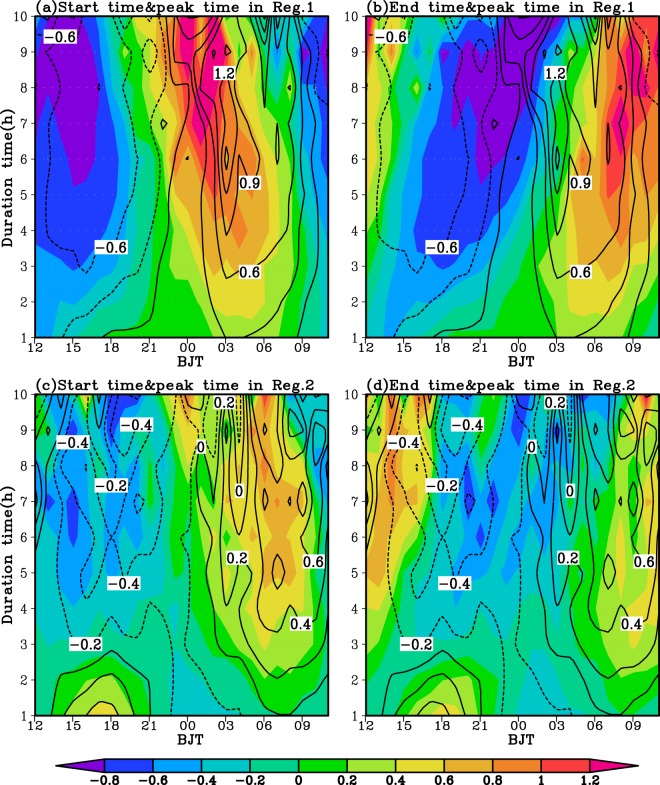
The diurnal cycle of the normalized occurrence frequency at the start and end (shading) and peak (contour) time for the EP events with different duration time over the Sichuan Basin (**a**,**b**) and the lower reaches of Yangtze River Basin (**c**,**d**).

To display the diurnal variation of occurrences of EP events with different duration much more clearly and intuitively, Fig. 7 further gives the normalized EPF at the start, peak and end time of EP events with different duration over each sub-region. Over the Sichuan Basin, the EP events with different duration frequently begin and peak at midnight to early morning and mostly end at 07:00–10:00 Beijing time. Meanwhile, the EP starts earlier and ends later with the duration time increased (Fig. 7a,b). The frequent occurrences of nocturnal EP events over the Sichuan Basin are closely related to the eastward-propagating convective systems originated over the Tibetan Plateau and the upward branch of mountain-plains solenoid induced by the differential heating between the Tibetan Plateau and Sichuan Basin^43^. As shown in Fig. 7c,d, the EP events with short (long) duration over the lower reaches of YRB mostly start and peak in the afternoon (morning) and typically end at 19:00–20:00 (around 09:00–14:00) Beijing time. The frequent occurrences of morning (afternoon) EP events over the lower reaches of YRB are initiated or enhanced by a nocturnal low level southwesterly winds (closely related to the local surface radiative heating)^41,43^. In addition, the EP events with short duration start and end later with the duration time increased. However, the EP events with long duration start earlier and end later with the duration time increased (Fig. 7c,d). Moreover, the long duration EP clearly tends to occur about 1–4 hours later over the lower reaches of YRB than over the Sichuan Basin in terms of starts, peaks and ends (Figs. 6 and 7).

### Diurnal variation of EP in different stages of warm season

**Figure 8:**
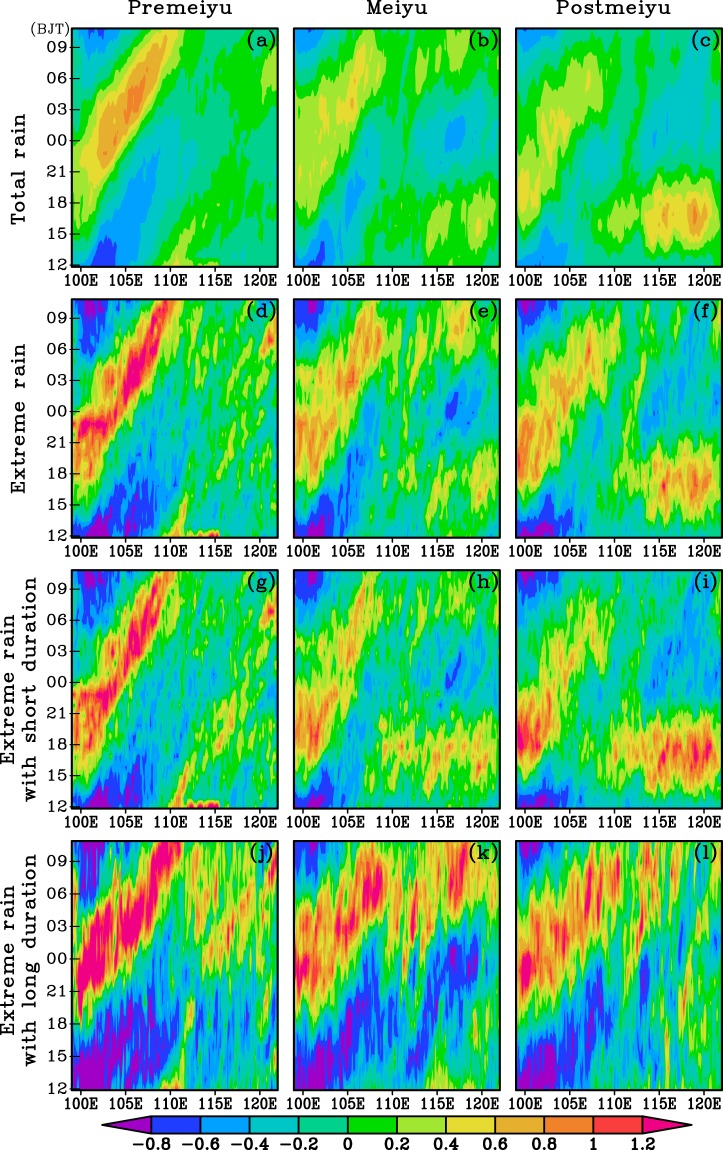
The time-longitude distribution of the normalized PA for the total precipitation (**a**,**b**,**c**), the total EP (**d**,**e**,**f**), and the EP with short (**g**,**h**,**i**) and long (**j**,**k**,**l**) duration during different periods of warm season over 2008–2016 regionally averaged along 26–34 ^o^N.

Figure 8 shows the propagation patterns of total PA, TEPA, and EPA with short and long duration during different stages of warm season. In the pre-Meiyu period, the total PA exhibits strong eastward propagating features over the regions between 100°E and 112°E. The precipitation systems form in the afternoon over the plateau and start to move eastward at around 18:00 Beijing time and end around noon of next day (Fig. 8a). During the Meiyu period, the precipitation systems developed over the Tibetan Plateau start to propagate eastward to the area near 110°E around 18:00 Beijing time and end around 09:00 Beijing time in the next day. Meanwhile, the PA over the regions east to 113°E in Meiyu period shows two comparable diurnal peaks with one originating around 113°E at 06:00 Beijing time and propagating eastward to 120°E around noon and the other stationary PA peak in the afternoon (Fig. 8b). In the post-Meiyu period, the eastward propagating systems initiated along the eastern Tibet can only proceed as far as 108°E and almost cease their activity in the next morning (Fig. 8c). Meanwhile, a stationary afternoon PA peaks is located over regions east to 110°E. The TEPA shows very similar pattern to the total PA during each stage of the warm season (Fig. 8d–f), indicating that the PA diurnal variations in warm season are largely determined by the EP events. The EPA for the short duration (Fig. 8g–i) also shows an eastward propagation with the same manner as the TEPA. The long duration EPA in the three stages of warm season shows very similar patterns over the regions west to110°E (Fig. 8j–l). The obvious eastward propagation of long duration EPA over the areas between 110°E and 118°E in morning can also be noted during Meiyu period.

**Figure 9:**
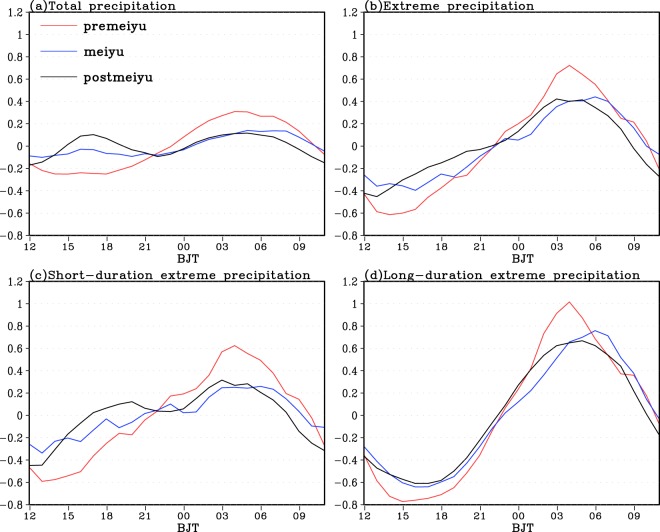
The diurnal variation of the normalized PF for the total precipitation (**a**), the total EP (**b**), and the EP with short (**c**) and long (**d**) duration over Sichuan Basin averaged over 2008–2016 during different periods of warm season.

As shown by Fig. 9a, the total PF in the Sichuan Basin during the pre-Meiyu period only shows one diurnal peak, which occurs at 04:00-05:00 Beijing time and is accompanied by the largest diurnal variability among the three periods in warm season. The peak occurs about 4 hours later during the Meiyu period with much smaller diurnal variability compared to the pre-Meiyu period. In the post-Meiyu period, the total PF exhibits two comparable diurnal peaks with one at around 04:00 Beijing time and the other located around 17:00 Beijing time. As shown in Fig. 9b, the TEPF during the three stages of warm season shows similar diurnal variation with one diurnal peak at around 03:00–06:00 Beijing time to the total PF. Among the three periods of warm season, the TEPF shows the largest diurnal peak located at 04:00 Beijing time in pre-Meiyu period and comparable diurnal peaks in early morning during Meiyu and post-Meiyu periods. The TEPF diurnal peak happens about 2 hours later (1 hour earlier) in Meiyu (post-Meiyu) period than in pre-Meiyu period. Similar to the total PF and TEPF, the short duration EPF (Fig. 9c) also displays the largest diurnal variability during pre-Meiyu period among the three stages of warm season with a diurnal peak in early morning. Meanwhile, the afternoon peak of short duration EPF becomes much more obvious during the post-Meiyu period relative to those during the pre-Meiyu and Meiyu periods. From Fig. 9b,d, the long duration EPF shows very similar diurnal cycles to the TEPF with an early morning diurnal peak during each stage but with much larger diurnal variability. Overall, the TEPF early morning peak over Sichuan Basin during each period of warm season is strongly determined by both short and long duration EP events.

**Figure 10:**
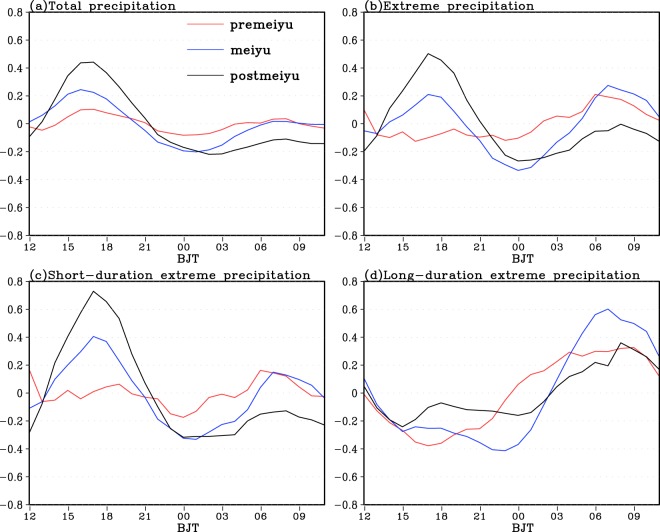
The diurnal variation of the normalized PF for the total precipitation (**a**), the total EP (**b**), and the EP with short (**c**) and long (**d**) duration over the lower reaches of Yangtze River Basin averaged over 2008–2016 during different periods of warm season.

The total PF over the lower reaches of YRB during the three periods of warm season typically shows two diurnal peaks with the predominant one in afternoon and the secondary one in morning (Fig. 10a), this is obviously different from the PF diurnal variation over Sichuan Basin (Fig. 9a). Meanwhile, the PF shows the smallest (largest) diurnal variability during Meiyu (post-Meiyu) period among the three stages of warm season. In addition, the afternoon (morning) PF diurnal peak becomes much more predominant (much weaker) during the post-Meiyu relative to the other two stages in the warm season (Fig. 10a). The TEPF during the Meiyu (post-Meiyu) period shows two comparable diurnal peaks with one in afternoon and the other in morning (a predominant afternoon peak and a secondary morning peak) (Fig. 10b), but the TEPF exhibits only one morning peak during the pre-Meiyu period, this is very different from the total PF shown in Fig. 10a. The short duration EPF in each stage of the warm season displays similar diurnal variation to that of the TEPF except that a dominant afternoon peak is observed during the Meiyu period (Fig. 10c). However, the long duration EPF during the three stages of warm season consistently shows a dominant morning peak (Fig. 10d). Overall, the short duration EP events largely contribute to the TEPF afternoon peaks over the lower reaches of YRB during the Meiyu and post-Meiyu periods, while both short and long duration EP events determine the TEPF morning peaks during each stage of warm season.

## Summery and discussion

Based on the hourly gauge-satellite merged precipitation data with the horizontal resolution of 0.1° × 0.1° during 2008–2016, the characteristics of EP diurnal cycle along the Yangtze River Basin (YRB) and their regional and sub-seasonal differences during warm season in terms of the EP start, peak and end time, and duration have been studied and indicated. Main findings are shown as follows:

The EPA along the YRB in warm season displays obvious diurnal variation which varies regionally: the EPA over most eastern Tibetan Plateau (Sichuan Basin and northern Yunnan-Guizhou Plateau) generally shows late afternoon to midnight (midnight to early morning) diurnal peaks. While the EPA over most lower reaches of YRB exhibits two diurnal peaks with one in late afternoon and the other in morning resulted from the short and long duration EP events, respectively. However, both short and long duration EP events lead to the nocturnal diurnal peaks and eastward propagating features of EPA over the regions west to110°E. The EP events over Sichuan Basin frequently start and peak in midnight to early morning and generally end at round 08:00 Beijing time, and they start earlier and end later with the duration time increased. While the EP events over the lower reaches of YRB with short (long) duration mostly begin and peak in afternoon (early morning) and typically end at around 18:00 (07:00-08:00) Beijing time, and they start (earlier) and end later with the duration time increased.

The EPF over the lower reaches of YRB (Sichuan Basin) shows obvious (much smaller) sub-seasonal differences in the diurnal phase. Over the lower reaches of YRB, the TEPF during Meiyu (post-Meiyu) period shows two comparable diurnal peaks with one in afternoon and the other in morning (a predominant afternoon peak and a secondary morning peak). While only one dominant morning TEPF peak is observed during the pre-Meiyu period. The TEPF afternoon (morning) peaks during the Meiyu and post-Meiyu periods (the three periods of warm season) are mainly resulted from the short (both short and long) duration EP events. Over the Sichuan Basin, the TEPF during each stage of warm season shows similar diurnal phase with only one early morning peak, which is strongly determined by both short and long duration EP events.

In current study, we have revealed the detailed characteristics of EP diurnal cycle along the YRB and compared their regional and sub-seasonal differences over Sichuan Basin and lower reaches of YRB. But what causes the difference is still an open question. Previous studies indicate that the complex terrain and monsoon circulation mainly contribute to the differences of EP diurnal cycle^21,34,45,47^. Firstly, from east to west along the YRB, there is obvious difference of terrain height between middle and upper reaches of YRB and lower reaches of YRB, especially in east Tibetan Plateau and Sichuan Basin covered by complex terrain. On the one hand, the heterogeneous heating between the mountain and basin causes the mountain-valley wind circulation, which plays a key role in the difference of diurnal peak time between mountains and basins^48,49^. Just like mountain-valley wind circulation, the sea-land breeze circulation results in the EP diurnal cycle over the coastal regions^50,51^. On the other hand, the eastward movement of convection systems and low vortex affected by the heating over the Tibetan Plateau are well related to the EP diurnal cycle along the YRB, especially the nocturnal EP events over the Sichuan Basin^29,43,52^. Secondly, the East Asian summer monsoon (EASM) dominates the summer precipitation change over the middle and lower reaches of YRB, the large-scale circulations of EASM systems, such as subtropical westerly jet, western Pacific subtropical high, both the intraseasonal variations of their position and strength lead to the sub-seasonal changes in the EP diurnal cycle^43,53,54^. Meanwhile, the mesoscale convective systems and tropical cyclone strengthen the EP diurnal cycle^55,56^. Some studies indicate that the terrain is well related to the short duration EP events^47^, and the low tropospheric circulation contributes to the long duration EP events^41^.

Although previous studies have revealed the possible mechanism between terrain and monsoon circulation of the EP diurnal cycle along the YRB, several questions are still unclear. For example, the precipitation well depends on the elevation^57^. In this study we only compare the EP diurnal characteristics between eastern Tibetan Plateau, the Sichuan Basin and northern Yunnan-Guizhou Plateau, but do not discuss the dependence of EP diurnal cycle on the elevation along the eastern slope of the Tibetan Plateau. Moreover, the precipitation in the Sichuan Basin is modulated by both the EASM and the circulations over the Tibetan Plateau^21,58,59^. Then what are the concurrent effects of two large-scale circulations on the EP diurnal cycle in Sichuan Basin? Finally, the thermal contrast between the Tibetan Plateau and western Pacific can influence the intraseasonal movement of EASM circulation systems and then modulate the diurnal cycle of precipitation^60,61^. In the future study, based on much finer spatiotemporal resolution data and numerical simulations, we will focus on examining the concurrent and individual effect of complex terrain and EASM on the EP diurnal cycle, and the contribution of thermal forcing over the Tibetan Plateau and western Pacific to the intraseasonal movement of EASM circulation system and EP diurnal cycle also should be discussed.
